# Site occupation and range expansion by the endangered, Mexican microendemic San Quintín Kangaroo Rat (*Dipodomys gravipes*)

**DOI:** 10.1093/jmammal/gyad113

**Published:** 2023-12-22

**Authors:** Jorge Andrade-Sánchez, Eric Mellink, Mónica E Riojas-López, Scott Tremor, Sula E Vanderplank

**Affiliations:** Departamento de Biología de la Conservación, Centro de Investigación Científica y de Educación Superior de Ensenada, Carretera Ensenada-Tijuana #3918, 22860 Ensenada, B.C., México; Departamento de Biología de la Conservación, Centro de Investigación Científica y de Educación Superior de Ensenada, Carretera Ensenada-Tijuana #3918, 22860 Ensenada, B.C., México; Departamento de Biología de la Conservación, Centro de Investigación Científica y de Educación Superior de Ensenada, Carretera Ensenada-Tijuana #3918, 22860 Ensenada, B.C., México; Departamento de Ecología, Centro Universitario de Ciencias Biológicas y Agropecuarias, Universidad de Guadalajara, Ramón Padilla Sánchez #2100, Zapopan, Jalisco, México; San Diego Natural and History Museum, P.O. Box 121390, San Diego, CA 92112, United States; Eco-Alianza de Loreto AC, 3419 Via Lido, Ste. 402, Newport Beach, CA 92663, United States

**Keywords:** agriculture, Baja California, farmland, Mammalia, Rodentia, agricultura, Baja California, tierras de cultivo, Mammalia: Rodentia

## Abstract

The San Quintin Kangaroo Rat, a rodent species microendemic to the San Quintin–El Rosario region in Baja California that was considered potentially extinct in the wild, was recently rediscovered. This stimulated subsequent searches by us throughout its known distribution range and on sites that seemed suitable beyond its limits. We captured the species at 19 out of 42 localities surveyed, of which 6 are beyond its historically known distribution range, expanding the latter by ~60 km. Most sites occupied by the species occur on abandoned farmland in early ecological successional stages. Our data support that in the highly transformed agricultural landscape into which the region was converted in the 20th century, the species was able to survive undetected and colonize/recolonize sites once habitat became adequate after agricultural abandonment. This exhibits that the species is highly resilient and persisted as a metapopulation. Further research and conservation actions must be framed within context of the region’s agricultural development.

## Teaser

The San Quintín Kangaroo Rat is highly resilient despite the severe decline of its population caused by agriculture in the 1970s and 1980s and is favored by farmland abandonment in early successional stages. Both suppression of riverine barriers and abandonment of farmed patches favored dispersal and establishment of the species in unoccupied patches beyond previous limits of its distribution.

## Resumen

Quintín–El Rosario, Baja California, considerada potencialmente extinta, se redescubrió recientemente. Ello estimuló búsquedas en todo su rango de distribución conocido y en sitios potenciales que parecían adecuados más allá de sus límites geográficos. Capturamos a la especie en 19 de 42 localidades, de las cuales 6 estaban fuera de su rango de distribución históricamente conocido, expandiendo este rango en ~60 km. La mayoría de los sitios ocupados por la especie se encuentran en tierras de cultivo abandonadas en etapas tempranas de sucesión ecológica. Nuestros datos respaldan la hipótesis de que el paisaje agrícola altamente antropizado, en el que se convirtió la región en el siglo XX, la especie sobrevivió en refugios y colonizó/recolonizó sitios una vez que el hábitat se volvió adecuado después de su abandono agrícola. Esto mostró que la especie es altamente resiliente y persistió como una metapoblación. Las futuras acciones de investigación y conservación deben enmarcarse en el contexto del desarrollo agrícola de la región.

**Fig. 1.**
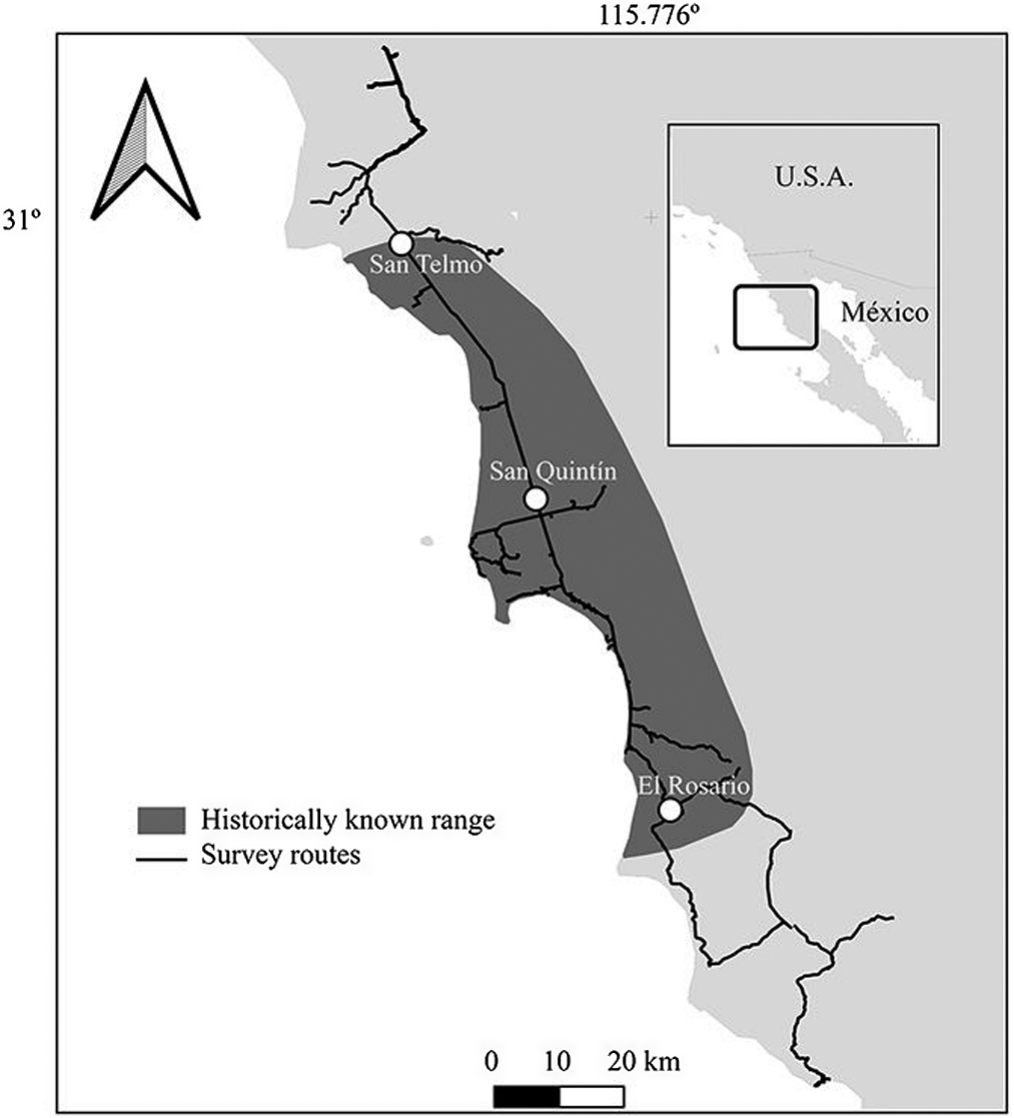
Known distribution of the San Quintín Kangaroo Rat (dark shade), and main search routes used to survey for its presence.

The San Quintin Kangaroo Rat (*Dipodomys gravipes*) is a “…large-sized, heavy-bodied, small-eared kangaroo rat, with a thick tail of medium length, the tip of tail dark, 5 toes on the extremely large-boned hindfoot” (Huey 1925:83). It is microendemic to the San Quintín coastal plain in Baja California—from San Telmo in the north to El Rosario in the south (Huey 1964; Best and Lackey 1985; Fig. 1). The eastern extent of the range is limited by thicker vegetation, steep slopes, and rockier soils. The first specimens of this species were collected in 1925 at the mouth of the Santo Domingo River canyon, and the species was described from them (Huey 1925). Validity of the species was later confirmed by karyotypic (Stock 1974), morphological (Schnell et al. 1978; Best 1981), and molecular analysis (Andrade-Sánchez et al. 2023).

The historic range of the San Quintín Kangaroo Rat covered approximately 1,000 km^2^. North of the El Rosario mountain range, the species occupied flat areas with friable soils that are found within coastal plains and adjacent rolling hills and mesas and are covered with low open Californian coastal sage scrub (sensu Kirkpatrick and Hutchinson 1977)—and to the south was found in the Arroyo El Rosario floodplain (Best 1983). The area occupied by the San Quintín Kangaroo Rat was prime agricultural land that after 1970 was almost entirely transformed to intensive farmland (Riemann 2015). Consequently, the species declined dramatically in abundance (Best 1983), and after 1,000 trap-nights north of San Quintín in July 1980 only 2 individuals (Best 1983) were detected. The species was last recorded in 1986 (Álvarez-Castañeda and Lacher 2018), although the locality was not indicated, and searches in the 1990s failed to find the species (Mellink 1996). Based on lack of captures and scale of land change, specialists considered it possibly extinct (Ceballos and Navarro 1991; Mellink 1996; Mellink and Luévano 2005), and the Mexican government listed it as “potentially extinct in the wild” in 2010 (SEMARNAT 2010). The International Union of Conservation of Nature considers it critically endangered (Álvarez-Castañeda and Lacher 2018).

After over 25 years since the last recorded observation, the San Quintín Kangaroo Rat was rediscovered in 2017 (Tremor et al. 2019). At that time, 4 individuals were captured on a ~3,000 m^2^ embankment surrounded by cropland, 5.6 km east of the town of San Quintín. This stimulated further searches by us throughout its known historic range, as well as on sites that seemed suitable to the north and south of its recorded range limits. Based on the results of these surveys, we present the current distribution of the species, gross habitat characteristics, and hypothesize on processes and drivers involved in its site occupation dynamics.

## Materials and methods

### Study area

We searched for the San Quintín Kangaroo Rat through its known historic range, as well as ~60 km farther to the north and ~50 km farther to the south (Fig. 1). Most of the area on Sierra El Rosario and to the north is within the southern extreme of the California Floristic Province—1 of 35 world biodiversity hotspots (Mittermeier et al. 2011). Our study area south of Sierra El Rosario is part of the Vizcaino Desert Province, a transitional zone that has vegetation characterizing both California Mediterranean and Sonoran Desert ecoregions.

**Fig. 2.**
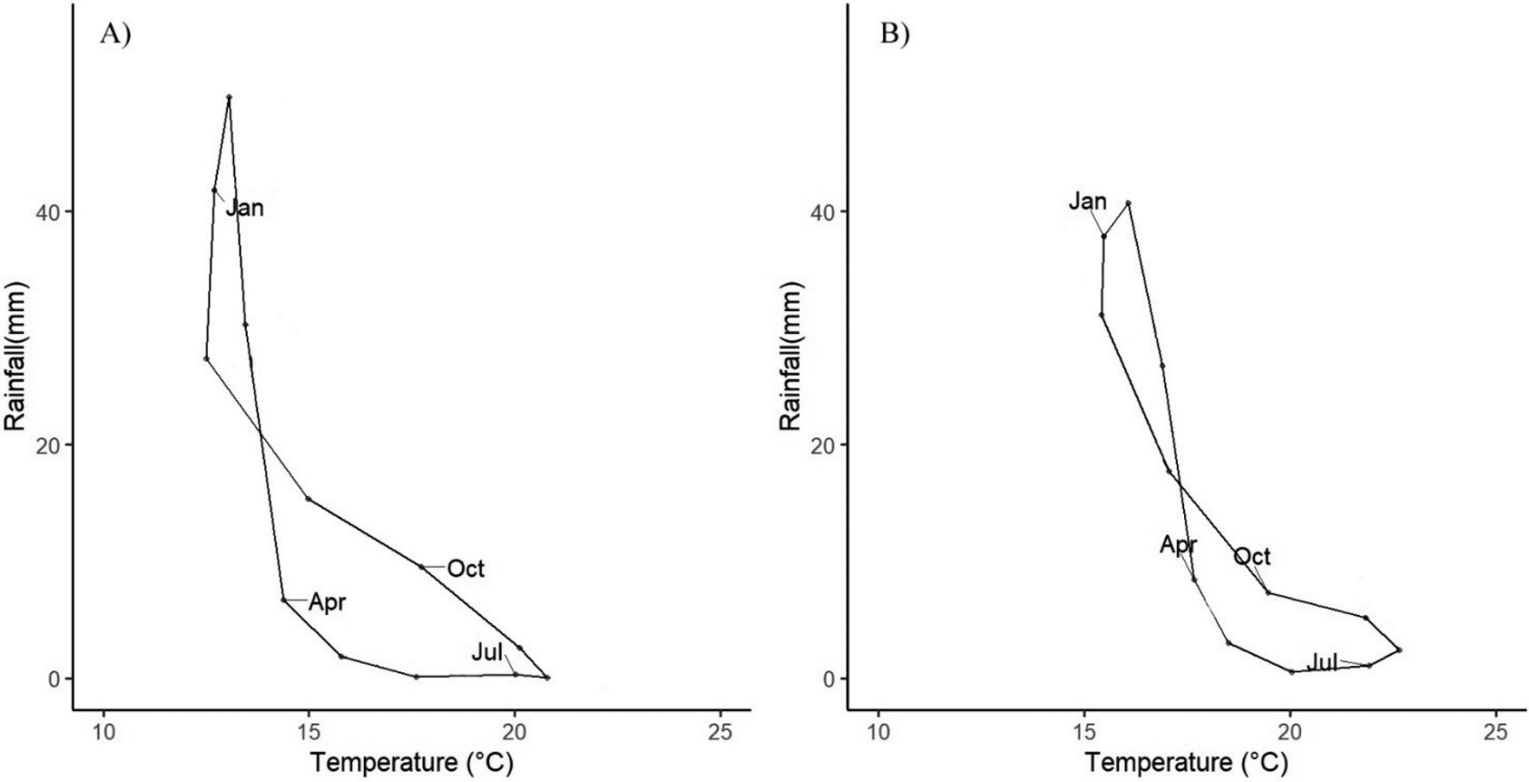
Climographs of the northern part of the San Quintín coastal plain (A) and El Rosario region (B), Baja California. Figures drawn from data in http://clicom-mex-cicese.mx.

Climate in our study area ranges from drier (119 mm average annual precipitation) and warmer (18.6 °C average annual temperature) in the south, to wetter (152 mm average annual precipitation) and colder (16.2 °C average annual temperature) in the north. Maximum temperatures in summer are 29.5 °C and 26.4 °C, respectively, and in the winter 22.4 °C and 19.6 °C (http://clicom-mex.cicese.mx). Minimum temperatures are 15.8 °C and 15.0 °C in summer, and 8.2 °C and 5.0 °C in winter, respectively. All rainfall occurs in the winter (Fig. 2).

### Field work

From 2017 to 2021 we surveyed all historical localities that were not actively farmed or urbanized, based on the San Diego Natural History Museum database, Lawrence M. Huey’s field notes, Vertnet (www.vernet.org), and the Global Biodiversity Information Facility (GBIF; www.gbif.org). We searched for potentially occupied sites within and beyond the historically documented distribution range of the species based on habitat that had been occupied (Best and Lackey 1985). At each promising site—both historical and potential—we looked for their distinctive round burrow entrances ≥8.4 cm high and ≥7.6 cm wide (Best 1976) and long runways. If we found sign of apparent presence of the species, we placed 3 Sherman traps (30.5 cm long) baited with rolled oats at the entrance of every active burrow and left them open from dusk to dawn. Our purpose was to confirm presence of the species, and 1 night of trapping was sufficient to do so. Total number of traps deployed at each site depended on number of active burrows. Additionally, in 2023 we scouted habitat along the dirt road to Puerto Santa Catarina and surroundings but did not find evidence of potential presence of the species.

We identified all kangaroo rats captured to species level based on external characteristics. Although the species is similar to the Dulzura Kangaroo Rat (*D. simulans*), body measurements are highly reliable to diagnose adults of both species (Andrade-Sánchez et al. 2023). We obtained body weight as well as total, tail, hindfoot, and ear length.

The area sampled at each survey site was determined by the surface occupied by active burrows. At each survey site, we recorded the most abundant plant species, identified according to our knowledge of regional flora and using the field guide of Rebman and Roberts (2012). We qualitatively estimated the proportion of soil covered by herbs and shrubs across each area sampled. Sites were assigned to land use categories of (i) no evidence of previous farming; (ii) abandoned farmland; (iii) urban; and (iv) garbage dump. We considered a site as “abandoned farmland” if original vegetation had been removed and old furrows were visible. We also noted land use in areas surrounding each colony.

We obtained elevation (m asl), slope (°), and terrain ruggedness values (Riley et al. 1999) from the 30 × 30 m ASTER Global Digital Elevation Model Ver. 3 at NASA’s Land Processes (https://asterweb.jpl.nasa.gov/gdem.asp). For all calculations, we used the Raster Terrain Analysis tools and the raster calculator of Quantum GIS (QGIS; Version 3.8.0; Zanzibar). Additionally, soil classification of all sites was extracted from Mexico’s national continuum soil spatial layer at a scale of 1:250,000 (INEGI 2016), using the Point Sampling tool of QGIS.

The proportion of cases that did not show evidence of previous farming, and of those classified as abandoned farmland were compared between sites currently occupied by the species versus those not occupied by the species through a χ^2^ test. A χ^2^ test was also utilized to determine whether type of habitat adjacent to the sites surveyed influenced presence or not of the species. We used an alpha level of *P* = 0.10 for all statistical tests.

### Ethical standards

We conducted this study in agreement with the Mexican environmental law (permits SGPA/DGVX/3150/19 and SGPA/DGVX/3150/22) and guidelines in Sikes et al. (2016).

## Results

**Fig. 3.**
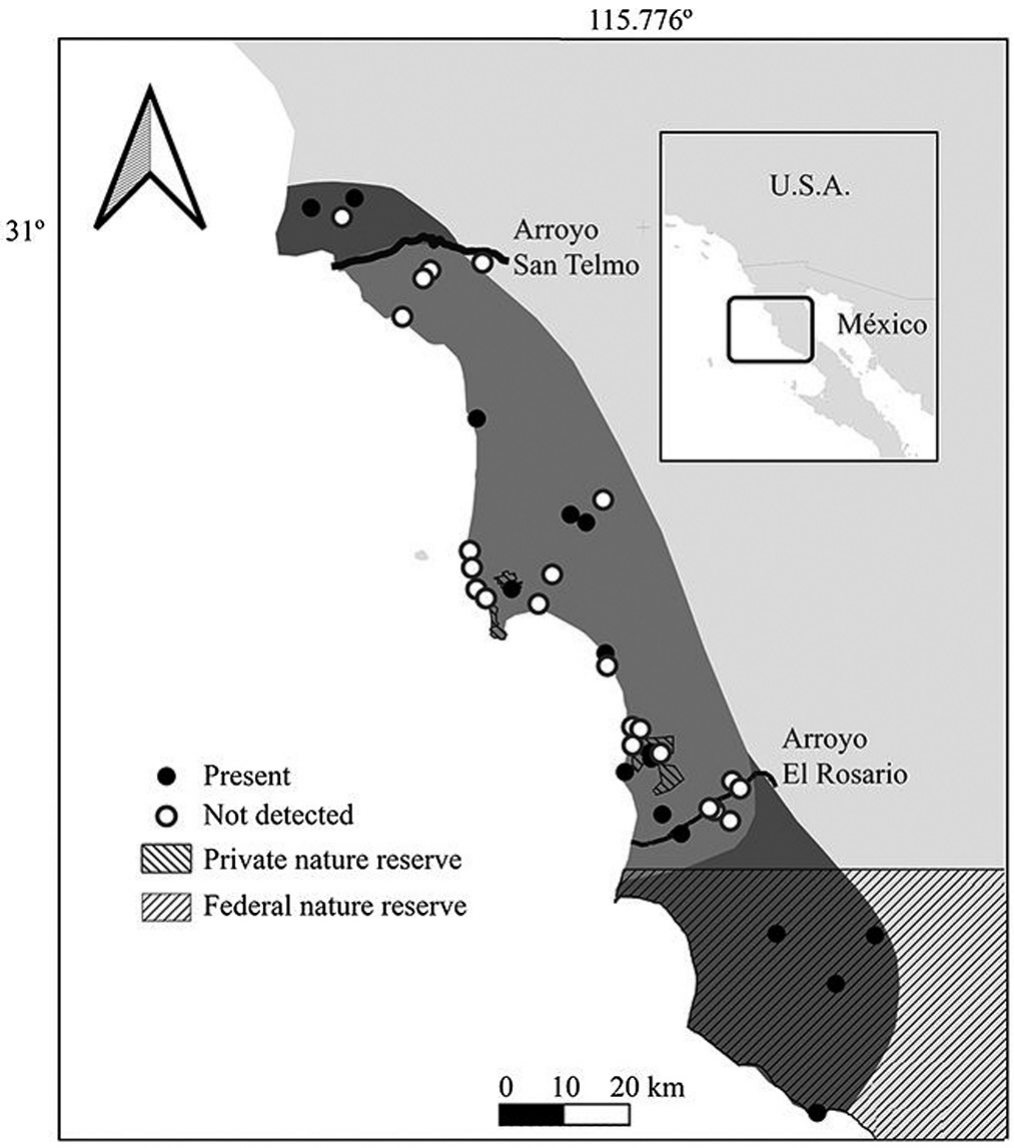
The San Quintín–Rosario area, Baja California, displaying sites where the San Quintín Kangaroo Rat was captured, and sites with no captures between 2017 and 2022.

Our trapping effort totaled ~2,400 trap-nights on 42 sites—34 within and 8 beyond the known historical distribution. We captured a total of 83 San Quintín kangaroo rats at 13 sites within and 6 beyond the known distribution (Fig. 3). The species was not captured at 23 of the sites surveyed. Of the 13 localities on which the species was captured within its historical distribution, 5 were localities at which it had been captured before the 1970–1980s decline, and 8 were localities at which it was recorded for the first time in our study. No signs of activity nor individuals of the species were captured at 3 localities where it had been trapped before its population collapse. The species was captured beyond its known historical geographic distribution at 2 sites to the north and 4 to the south. During the scouting trip to Puerto Santa Catarina and its surroundings, we found no burrows of the species in the few open patches explored.

Locations at which the species was present either historically or currently (*n* = 22) had an elevation of 6–251 m asl, a slope of 0.7–8.5°, and a ruggedness value of 0.37–3.12. Soil type was fluvisol at 5, planosol at 3, regosol at 4, solonchak at 1, and xerosol at 10 locations. Sites that we considered potential and explored for the species presence but were we did not find signs of it (*n* = 19) had values of 7–128 m asl elevation, 0.5–9.7° slope, and 0.5–5.12 ruggedness—and soil types of fluvisol at 3, planosol at 1, regosol at 3, solonchak at 5, and xerosol at 7 locations. Eighty-four percent of the sites where we captured the species were abandoned farmland plots, including sites within the historic range as well as those in the expanded range, and 83% of historical locations where we captured the species were now abandoned farmland (Fig. 3, Table 1). The species tended to occupy abandoned farmland more commonly than sites without evidence of previous farming (χ^2^ = 3.11, *P* = 0.08). There was no effect of adjacent habitat type on presence or absence of the species (Table 1; χ^2^ = 5.01, *P =* 0.41).

**Table 1.** Current condition and adjacent habitat of sites on which San Quintín Kangaroo Rats were captured and of sites at which they were not. Numbers in columns are the number of sites in each category. Baja California, Mexico, 2017–2022.

Case	Total	Current condition: % (number)			Adjacent habitat: % (number)					
		No farming evidence	Abandoned farmland	Garbage dump	Shrub patch	Coastal sage scrub	Dune vegetation	Marsh vegetation	Cropland	Crops & urban
Absent in historic range	23	75 (9)	45 (13)	100 (1)	73 (8)	25 (2)	50 (2)	75 (3)	54 (7)	4 (1)
Historic site	4	8 (1)	7 (2)	100 (1)	27 (3)	0	0	0	0	100 (1)
Potential site	19	67 (8)	38 (11)	0	46 (5)	25 (2)	50 (2)	75 (3)	54 (7)	0
Present	19	25 (3)	55 (16)	0	27 (3)	75 (6)	50 (2)	25 (1)	46 (6)	5 (1)
In historic range	6	8 (1)	17 (5)	0	9 (1)	0	50 (2)	25 (1)	8 (1)	100 (1)
New in historic range	7	8 (1)	21 (6)	0	0	50 (4)	0	0	23 (3)	0
New in north expansion	2	0	7 (2)	0	0	0	0	0	15 (2)	0
New in south expansion	4	8 (1)	10 (3)	0	18 (2)	25 (2)	0	0	0	0

The most common plant species found in survey sites—whether occupied or not by the species—were Vizcaíno Saltbush (*Atriplex julacea*), Menzie’s Goldenbush (*Isocoma menziesii*), Broom Baccharis (*Baccharis sarathoides*), and Crystalline Iceplant (*Mesembryanthemum crystallinum*). The last species—an invasive exotic—was present at all sites, whether they were or not occupied by the species (Supplementary Data SD1). The San Quintín Kangaroo Rat only used sites with <10% ground cover by plants, regardless of whether it was by herbaceous plants and an absence of shrubs (Table 2), except at 3 sites that had a few shrubs.

**Table 2.** Vegetation of sites on which San Quintín kangaroo rats were captured and of sites at which they were not. Numbers in columns are number of sites in each category.

Case	Total	Soil cover by plants: % (number)		Herbs: % (number)		Shrubs >0.05 m: % (number)	
		<10%	>10%	No or few	Yes	No or few	Yes
Absent in historic range	23	44 (15)	100 (8)	100 (8)	44 (15)	49 (18)	100 (5)
Historic site	4	9 (3)	12 (1)	37 (3)	3 (1)	8 (3)	20 (1)
Potential site	19	35 (12)	88 (7)	63 (5)	41 (14)	40 (15)	80 (4)
Present	19	56 (19)	0	0	56 (19)	51 (19)	0
In historic range	6	18 (6)	0	0	18 (6)	16 (6)	0
New in historic range	7	20 (7)	0	0	20 (7)	19 (7)	0
New in north expansion	2	6 (2)	0	0	6 (2)	5 (2)	0
New in south expansion	4	12 (4)	0	0	12 (4)	11 (4)	0

## Discussion

Our results not only document presence of the San Quintín Kangaroo Rat throughout its known historical distribution, but also extend it ~10 km to the north and ~50 km to the south, surpassing 2 historically active watercourses—Arroyo San Telmo and Arroyo El Rosario—that had been considered as possible barriers to dispersal (Fig. 3, Table 1). Our research endorses the 2017 rediscovery of the species (Tremor et al. 2019) and allows for a general description of habitat characteristics of sites colonized or recolonized by the species.

**Fig. 4.**
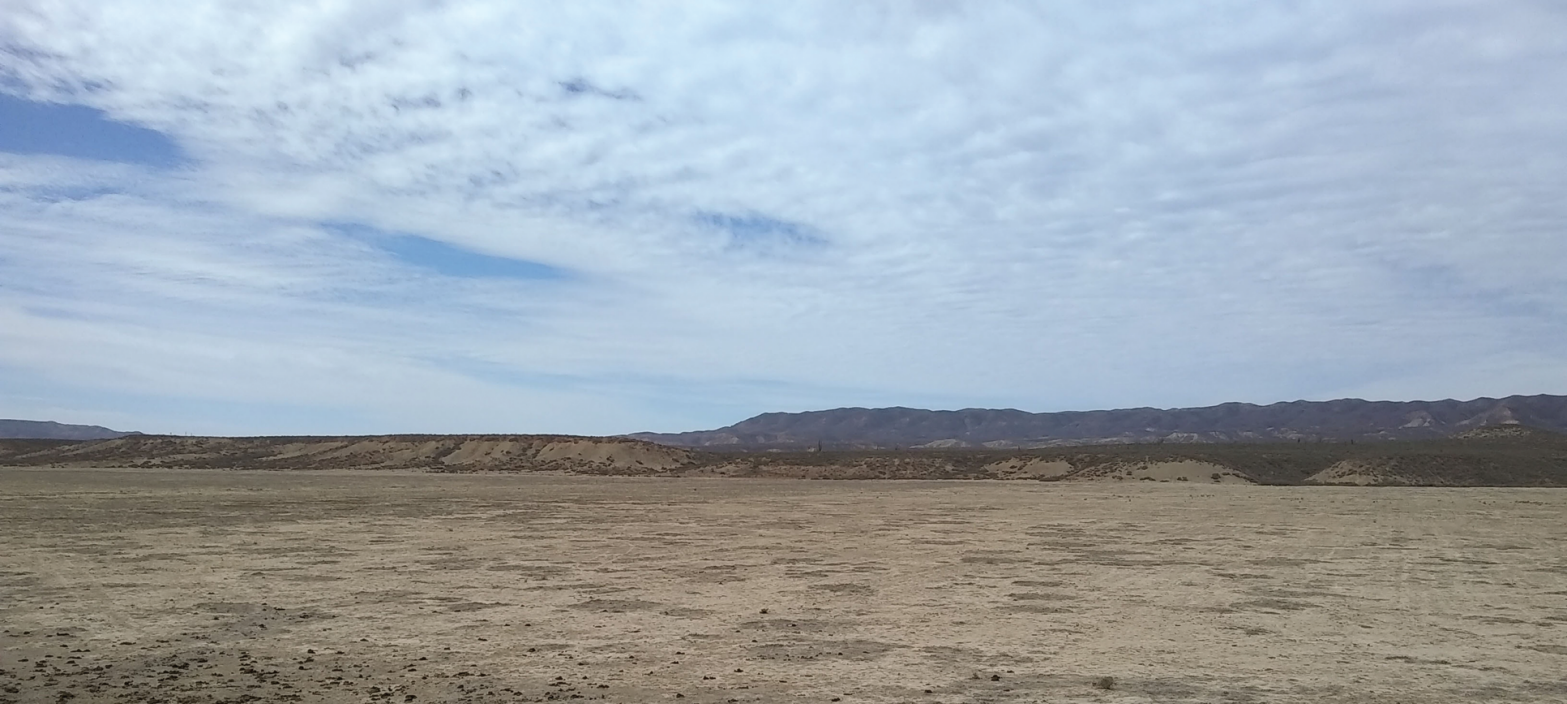
Typical habitat of the San Quintin Kangaroo Rat, showing the flat area with low cover by herbaceous vegetation at San Vicentito, an abandoned farmland.

The San Quintin Kangaroo Rat has been considered sensitive to habitat disturbance, and agricultural conversion was identified as a major driver of its population decline in the 1970s and 1980s (Best 1983; Best and Lackey 1985; Lidicker 1989; Cab-Sulub and Álvarez-Castañeda 2020). Our results complement the picture of the relationship of the species with agricultural conversion in that abandoned farmland can provide suitable habitat for the species, where we documented its occurrence on 55% of abandoned farmland plots that we surveyed, including 83% of all historical sites in our study where the species was present (5 out of 6), and in 86% of the new localities here reported (Table 1; Fig. 4). Indeed, some places that were unsuitable when covered with their original habitat now provide suitable habitat for the species after being cleared for farming and later abandoned—colonized after persisting in refugia during extensive farming of the San Quintín plain and El Rosario riverbed. This capability to colonize farmland after cultivation has been reported also for a congener the Stephens’s Kangaroo Rat (*D. stephensi*; Thomas 1975; Moore-Craig 1984; Price and Endo 1989; US Fish and Wildlife Service 2020).

All locations at which we captured the species had <10% cover by herbs and few or no shrubs. This suggests that, in abandoned farmlands, the species uses sites in early successional stages. Those stages that had more than a few shrubs were not used by the species. In the region, sites in later stages include shrubs including Ragweed (*Ambrosia chenopodifolia*), Spiny Rush (*Juncus acutus*), Baja Desert-thorn (*Lycium brevipes*), and California Desert-thorn (*Lycium californicum*).

Differences between abandoned farmland plots occupied and those that were not occupied is not clear. The fact that there were no differences in physical characteristics of the terrain (soil type, slope, elevation, and ruggedness) between plots occupied and not occupied indicates that factors other than those considered herein could explain habitat selection. Our results confirm that the San Quintín Kangaroo Rat occupies flatland with scarce vegetation, as also described by other researchers (Best 1983; Best and Lackey 1985). However, in addition to avoidance of sites with >10% plant cover and sites with shrubs taller than 0.5 m, additional habitat characteristics remain to be investigated.

The 6 sites beyond the traditionally accepted distribution range where we documented the species (Fig. 2) represent an important extension of its known range. The northern limit accepted in the literature was that defined by Huey (1964) and neither he nor other mammalogists appear to have carried out collecting immediately north of the San Telmo river according to GBIF, Vernet, and Huey’s field notes.

Regarding the southern limit, Huey wrote in his field notes on 5 May 1925 “… I am satisfied that the large kangaroo rat - D. gravipes - found farther north does not occur this far south.” Documentation of specimens in 1966 by T. Best in the El Rosario riverbed (Vertnet) after Arroyo El Rosario had ceased to be a perennial stream and water flows were greatly reduced could indicate colonization of this area between those 2 dates. As with the northern limit, there are no known small mammal surveys on the coastal plain between Arroyo El Rosario and Punta San Carlos (Fig. 2) prior to ours, or their results were not made public as publications or as specimens. Although the species could have occurred in this area but unnoticed, the fact that all colonies south of Arroyo El Rosario are on abandoned farmland indicate that a more recent colonization is more likely. As our reconnaissance of the road to Puerto Santa Catarina revealed, Punta San Carlos is currently the southernmost location of the species.

In 2018, the International Union for the Conservation of Nature (IUCN) published a distribution map of the San Quintín Kangaroo Rat (Álvarez-Castañeda and Lacher 2018), without clarifying the procedure used to generate it. This map must be taken as a general approximation and not as a precise representation because: (i) range limits displayed were not supported by known records; and (ii) it includes a large area of mountainous terrain that is unsuitable for the species. The map was a good working approximation but must now be updated with information here presented.

This process of dispersal through a hostile agricultural matrix and colonization of abandoned farmland patches would have not been limited to the historical distribution range if previous barriers, if any, were removed. The species colonized patches outside the previous range after the lower sections of Arroyos San Telmo and El Rosario dried following ~4 decades of water extraction for human needs (Riemann 2015).

Whether the species survived in one or multiple refugia after the San Quintín plain was extensively transformed for agricultural production is not known at this time, but subsequent colonization and, or recolonization of patches in an ecological time frame indicates that the species is currently subject to metapopulation dynamics (Hanski and Gilpin 1991). Analyzing spatial patterns of occurrence in more detail would all for an evaluation of structural and functional connectivity of populations and contribution of each patch for persistence, providing a foundation for informed spatially based conservation planning for the species. Based on our data, we hypothesize that abandoned farmland in early ecological succession enhances colonization by the species. However, as shrubs encroach upon colonies the species ceases to use those patches. The current patchy spatial distribution of populations is largely due to anthropogenic activities—hence, further research and conservation actions must be framed within the context of agricultural development, as well as under a metapopulation approach.

## Supplementary data

Supplementary data are available at *Journal of Mammalogy* online.

**Supplementary Data SD1.**—Physical characteristics of the site.

gyad113_suppl_Supplementary_Datas_SD1

## Data Availability

Data are available at Open Science Framework Repository: https://osf.io/eq8mv/.
