# Supplementary material for: Site occupation and range expansion by the endangered, Mexican microendemic San Quintín Kangaroo Rat (Dipodomys gravipes)
Source: J Mammal. 2023 Dec 22;105(1):168–74. doi: 10.1093/jmammal/gyad113 (PMC10894624; doi:10.1093/jmammal/gyad113)
Supplement: gyad113_suppl_Supplementary_Datas_SD1 [file gyad113_suppl_supplementary_datas_sd1.docx]

Supplementary Data SD1

| Locality | Latitude | Longitud | Capture | Condition | History | Adjacency |
| --- | --- | --- | --- | --- | --- | --- |
| Jaramillo | 30.9195 | -116.1105 | 1 | Fallow | Historic | Crops |
| San Ramón | 30.7015 | -116.0272 | 3 | Fallow | Historic | Crops-Urban |
| Agua Chiquita | 30.5399 | -115.8571 | 17 | Fallow | Historic | Shrubs |
| Punta Azufre | 30.411 | -115.9296 | 3 | Never farmed | Historic | Marsh |
| El Socorro | 30.3365 | -115.8271 | 4 | Fallow | Historic | Dune |
| San Jacinto | 31.0446 | -116.2175 | 1 | Fallow | New | Crops |
| Mesa Agua Chiquita | 30.5522 | -115.8814 | 2 | Fallow | New | Crops |
| Monte Ceniza | 30.4362 | -115.9731 | 6 | Fallow | New | Sage |
| Valle Tranquilo RN | 30.1825 | -115.7564 | 2 | Fallow | New | Sage |
| El Campito | 30.1517 | -115.7968 | 2 | Fallow | New | Dune |
| Mesa militares | 30.0857 | -115.7387 | 7 | Fallow | New | Sage |
| Parcela 91 | 30.1724 | -115.7558 | 1 | Never farmed | New | Sage |
| Mesa Sur | 30.0556 | -115.7095 | 2 | Fallow | New | Crops |
| Colonet | 31.0294 | -116.285 | 5 | Fallow | North | Crops |
| Arroyo San Telmo | 31.0446 | -116.2175 | 3 | Fallow | North | Crops |
| San Vicentito | 29.9 | -115.561 | 7 | Fallow | South | Sage |
| Las Pintas | 29.8975 | -115.4078 | 4 | Fallow | South | Sage |
| Malbar | 29.822 | -115.4687 | 15 | Fallow | South | Shrubs |
| San Carlos | 29.6214 | -115.4976 | 3 | Never farmed | South | Shrubs |
| Arroyo San Telmo (a) | 31.0151 | -116.237 | 0 | Fallow | Potential | Crops |
| San Telmo | 30.944 | -116.0185 | 0 | Fallow | Potential | Crops |
| Jaramillo north | 30.9323 | -116.0994 | 0 | Fallow | Potential | Crops |
| Jaramillo east | 30.9195 | -116.1105 | 0 | Fallow | Potential | Crops |
| San Jacinto Sw | 30.8601 | -116.1429 | 0 | Fallow | Potential | Crops |
| Agua Amarga | 30.5752 | -115.8307 | 0 | Fallow | Potential | Shrubs |
| Desalination plant | 30.4958 | -116.0379 | 0 | Fallow | Potential | Sage |
| San Quintín S.R. | 30.4695 | -116.0346 | 0 | Never farmed | Potential | Sage |
| Punta Mazo | 30.4362 | -116.0276 | 0 | Never farmed | Potential | Dune |
| Punta Mazo dunes | 30.4219 | -116.0127 | 0 | Never farmed | Potential | Dune |
| San Simón | 30.4591 | -115.9099 | 0 | Never farmed | Potential | Shrubs |
| Punta Azufre south | 30.4135 | -115.9313 | 0 | Never farmed | Potential | Marsh |
| Socorrito | 30.317 | -115.8242 | 0 | Never farmed | Potential | Marsh |
| Arroyo hondo | 30.222 | -115.7854 | 0 | Never farmed | Potential | Shrubs |
| Onion fields | 30.2183 | -115.7728 | 0 | Fallow | Potential | Crops |
| Valle Tranquilo (out) | 30.1933 | -115.7848 | 0 | Fallow | Historic | Shrubs |
| Valle Tranquilo inland | 30.1809 | -115.7413 | 0 | Never farmed | Potential | Shrubs |
| Rancho Gonzalez | 30.1374 | -115.6303 | 0 | Fallow | Potential | Crops |
| Arroyo El Rosario | 30.1259 | -115.6175 | 0 | Fallow | Potential | Shrubs |
| Rosario dump | 30.0917 | -115.6572 | 0 | Urban | Potential | Urban |
| Rosario south | 30.0755 | -115.6331 | 0 | Never farmed | Historic | Shrubs |
| Panteón inglés | 30.4259 | -115.9356 | 0 | Fallow | Potential | Marsh |
| Rosario riverbed | 30.0957 | -115.6653 | 0 | Fallow | Historic | Shrubs |

| Locality | Cover | Slope | Herbs | Shrubs >0.5m | Soil | Slope | Ruggness | Elev |
| --- | --- | --- | --- | --- | --- | --- | --- | --- |
| Jaramillo | <10 | <10% | Yes | No | Xerosol | 2.52 | 1.63 | 72 |
| San Ramón | <10 | <10% | Yes | No | Xerosol | 0.78 | 0.38 | 7 |
| Agua Chiquita | <10 | <10% | Yes | No | Planosol | 5.25 | 2.38 | 60 |
| Punta Azufre | <10 | <10% | Yes | No | Solonchak | 1.44 | 1.25 | 6 |
| El Socorro | <10 | <10% | Yes | No | Xerosol | 3.44 | 1.75 | 9 |
| San Jacinto | <10 | <10% | Yes | No | Fluvisol | 6.17 | 2.50 | 28 |
| Mesa Agua Chiquita | <10 | <10% | Yes | No | Xerosol | 8.46 | 2.63 | 103 |
| Monte Ceniza | <10 | <10% | Yes | No | Regosol | 4.96 | 2.13 | 22 |
| Valle Tranquilo RN | <10 | <10% | Yes | No | Regosol | 3.50 | 2.50 | 96 |
| El Consuelo | <10 | <10% | Yes | No | Xerosol | 2.47 | 1.00 | 17 |
| Mesa militares | <10 | <10% | Yes | No | Planosol | 0.70 | 0.50 | 251 |
| Parcela 91 | <10 | <10% | Yes | Few | Regosol | 6.18 | 2.50 | 120 |
| Mesa Sur | <10 | <10% | Yes | No | Fluvisol | 4.86 | 1.75 | 49 |
| Colonet | <10 | <10% | Yes | No | Planosol | 2.24 | 2.75 | 82 |
| Arroyo San Telmo | <10 | <10% | Yes | No | Fluvisol | 6.17 | 2.50 | 28 |
| San Vicentito | <10 | <10% | Yes | No | Regosol | 8.09 | 3.13 | 147 |
| Las Pintas | <10 | <10% | Yes | Few | Xerosol | 0.78 | 2.25 | 234 |
| Malbar | <10 | <10% | Yes | Few | Xerosol | 4.21 | 1.63 | 153 |
| San Carlos | <10 | <10% | Yes | No | Xerosol | 7.38 | 3.00 | 21 |
| Arroyo San Telmo (a) | <10 | <10% | Yes | No | Fluvisol | 5.15 | 2.13 | 24 |
| San Telmo | <10 | <10% | Yes | No | Fluvisol | 4.54 | 1.88 | 128 |
| Jaramillo north | <10 | <10% | Yes | No | Xerosol | 9.71 | 1.88 | 128 |
| Jaramillo east | <10 | <10% | Yes | No | Xerosol | 2.52 | 1.63 | 72 |
| San Jacinto Sw | <10 | <10% | Yes | No | Xerosol | 1.11 | 1.50 | 10 |
| Agua Amarga | >10 | <10% | Few | No | Regosol | 3.51 | 1.88 | 108 |
| Desalination plant | <10 | <10% | Yes | No | Solonchak | 6.91 | 2.63 | 67 |
| San Quintín S.R. | >10 | <10% | Few | Yes | Solonchak | 1.11 | 2.00 | 13 |
| Punta Mazo | >10 | <10% | Yes | No | Solonchak | 3.51 | 1.88 | 14 |
| Punta Mazo dunes | >10 | <10% | Yes | No | Solonchak | 2.24 | 1.88 | 14 |
| San Simón | >10 | <10% | No | Yes | Xerosol | 4.08 | 2.13 | 14 |
| Punta Azufre south | <10 | <10% | Yes | No | Solonchak | 5.00 | 2.00 | 10 |
| Socorrito | <10 | <10% | Yes | No | Xerosol | 2.47 | 2.00 | 11 |
| Arroyo hondo | >10 | <10% | No | Yes | Xerosol | 6.94 | 3.00 | 47 |
| Onion fields | <10 | <10% | Yes | No | Planosol | 3.51 | 4.63 | 98 |
| Valle Tranquilo (out) | <10 | <10% | Yes | No | Xerosol | 1.98 | 1.75 | 43 |
| Valle Tranquilo inland | <10 | <10% | Yes | No | Regosol | 2.45 | 2.25 | 120 |
| Rancho Gonzalez | <10 | <10% | Yes | No | Regosol | 9.11 | 5.13 | 121 |
| Arroyo El Rosario | >10 | <10% | No | Yes | Fluvisol | 0.50 | 0.50 | 73 |
| Rosario dump | <10 | <10% | No | No | Fluvisol | 4.55 | 2.88 | 92 |
| Rosario south | <10 | <10% | No | No | Xerosol | 4.21 | 1.13 | 158 |
| Panteón inglés | <10 | <10% | Yes | No | Xerosol | 0.50 | 0.50 | 7 |
| Rosario riverbed | >10 | <10% | No | Yes | Fluvisol | 4.55 | 2.88 | 92 |
